# Secular trends in incidence of acute gastroenteritis in general practice, France, 1991 to 2015

**DOI:** 10.2807/1560-7917.ES.2017.22.50.17-00121

**Published:** 2017-12-14

**Authors:** Mathieu Rivière, Noémie Baroux, Vanina Bousquet, Katia Ambert-Balay, Pascal Beaudeau, Nathalie Jourdan-Da Silva, Dieter Van Cauteren, Frédéric Bounoure, Fanny Cahuzac, Thierry Blanchon, Thierry Prazuck, Clément Turbelin, Thomas Hanslik

**Affiliations:** 1 Sorbonne Universités, UPMC Univ Paris 06, INSERM, Institut Pierre Louis d’épidémiologie et de Santé Publique (IPLESP UMRS 1136), Paris, France; 2 Infectious disease department, CHR Orléans La Source, Orléans, France; 3 Santé publique France, the French national public health agency, Saint-Maurice, France; 4 National Reference Center for Gastroenteritis Viruses, Laboratory of Virology, CHU of Dijon, Dijon, France; 5 Pharmaceutics Laboratory (DC2N INSERM U982), Faculty of Medicine and Pharmacy, University of Rouen, Rouen, France; 6 Université Versailles-Saint-Quentin-en-Yvelines, Versailles, France

**Keywords:** gastrointestinal illness, sentinel surveillance, trend, France

## Abstract

We analysed 25 years of general practitioner (GP) visits for acute gastroenteritis (AG) surveillance in France, by the GP Sentinelles network. We searched for time trends of acute gastroenteritis incidence during winter periods. Data from emergency departments and drug reimbursement were additional data sources. A time-series analysis was performed using a generalised additive model for all data sources for the winter period. Virological data were incorporated and compared with the three data sources. The cumulative incidence of GP visits for winter AG exhibited an increasing trend from 1991 until 2008, when it reached 6,466 per 100,000 inhabitants. It decreased thereafter to 3,918 per 100,000 inhabitants in 2015. This decreasing trend was observed for all age groups and confirmed by the generalised additive model. For emergency department visits a decreasing trend was observed from 2004. Drug reimbursement data analyses demonstrated a decreasing trend from when data began in 2009. The incidence reported by GPs and emergency departments was lower following the emergence of norovirus GII.4 2012 (p < 0.0001). Winter AG incidences seem to follow long-term rising and decreasing trends that are important to monitor through continuous surveillance to evaluate the impact of prevention strategies, such as future immunisation against acute viral gastroenteritis.

## Introduction

Acute gastroenteritis (AG) is commonly defined as diarrhoea (three or more loose stools) or vomiting in the past 24 hours [1]. AG can be caused by different viruses, bacteria, and parasites, as well as chemicals and other non-infectious agents. The frequency of detection of infectious agents depends on the age of the patients and the epidemiological context [2]. In developed countries, although AG is generally a mild disease, its morbidity and economic burden are high [3,4]. In France, it has been estimated that more than 21 million AG cases occur each year, leading to between 1.5 and 4 million general practitioner (GP) visits by adults during the winter period [3,5]. AG occurs year-round but exhibits a pronounced winter peak, usually between December and March in France, related to an increase in AG of viral origin, mainly norovirus and rotavirus [6-8]. AG incidence is higher in children and rotavirus is the most common cause of severe AG in young children. Worldwide, it is estimated that rotavirus is responsible for more than 25 million clinic visits each year [9].

Although several countries have been collecting data on AG for many years, long-term temporal trends of AG incidence are still not well-known and are under-researched. Most time-series analyses focus on the impact of the rotavirus vaccination, showing a decreasing trend of rotavirus incidence and AG consultations for children since the introduction of vaccination programmes in several European countries [10]. A recent study in the United Kingdom (UK) showed a decrease in AG incidence of 26% and 23% in the < 1 and 1–4 years age groups, respectively, after the introduction of the vaccination programme in 2013 [11]. In the United States, the same trend was observed after the introduction of vaccination in 2006; the proportion of tests positive for rotavirus declined, ranging from 58% to 90% in each of the 7 post-vaccine reporting years, compared with the combined pre-vaccine years (2000–2006) [12]. However, studies have also shown a decreasing incidence of AG in the absence of the rotavirus vaccination programme. In the Netherlands, AG consultations for children under 5 years old and rotavirus detection both decreased in the period from January to April 2014, compared with the same period in previous years. This decrease was 36% for AG consultation and 72% for rotavirus detection [13].

Since 1990, the French GP Sentinelles network has conducted continuous surveillance of AG in France. Here, we report our analysis of the first 25 years of surveillance, with the aim of searching for time trends in the winter-to-winter season of AG incidence in France, where rotavirus vaccination is not included in the national immunisation programme [14].

## Methods

### Data sources

The French Sentinelles network is a nationwide network of more than1,300 GPs (2% of all GPs in France) involved in the surveillance of different health indicators and epidemiological studies. GPs participate on a voluntary basis and report data weekly via a secure Internet connection. Sentinelles GPs differ from other French GPs in terms of location, sex distribution and number of consultations, but estimated AG incidence rates are corrected for bias due to lack of representativeness by estimating the weighted incidence using external information for the medical population per region for each year [15]. This network has conducted AG surveillance since 1990 [16,17], in which AG is defined as cases with at least three watery or near-watery stools per day, lasting for fewer than 14 days. Each week, 400 GPs report the weekly number of visits for AG and describe individual patient characteristics (age, sex, location, hospitalisation if required). ‘GP visits’ refers to both GP visits and home visits. The Sentinelles network routinely provides weekly national incidence rates per 100,000 inhabitants, estimated as follows: the mean number of cases per Sentinelles GP (standardised according to their participation and their geographical distribution) is multiplied by the total number of GPs in France and the result is then divided by the population of that year, using the French population included in national censuses as a reference. The methodology to estimate the incidence of consultations for AG has been previously described [15]. Data for the 1991­–2015 period were used for the current analysis. For the rest of this paper, these data will be referred to as GP visit data.

Other data sources included data from hospital emergency departments (EDs) and from the national health insurance (NHI) database. A syndromic surveillance network of hospital emergency departments (EDs), called OSCOUR (Organisation de la Surveillance COrdonnée des URgences), has been coordinated by the French national public health agency since 2004 [18]. In 2015, the OSCOUR network coverage accounted for 88% of attendance in EDs nationally. Data are collected automatically daily from patients’ computerised medical files [18]. Age, sex, and diagnosis, coded according to the International Classification of Diseases, Tenth Revision (ICD-10) [19], are collected. ED visits for AG are defined as a visit with at least one code from A08 to A099. Weekly numbers of attendances for AG and for all causes with a diagnosis were analysed from 2005 to 2015. In this paper, these data will be referred as ‘ED visit data’.

The NHI database covers more than 98% of the population living in France and records all drug reimbursements and payments to professionals for consultations [20]. An algorithm aimed at discriminating AG prescriptions from non-AG prescriptions was built using patient characteristics, type and number of drugs prescribed, and number of boxes delivered, allowing the estimation of the duration of the treatment and the time lag between consultation and dispensation [21]. Weekly estimates of prescriptions for AG were analysed from 2010 to 2015. In this paper, these data will be referred to as drug reimbursement data.

Finally, data describing the predominant circulating strains of norovirus and group A rotavirus, which were the most frequent strains isolated in positive samples for each seasonal period, were included in our analyses. Identification of viral AG clusters is routinely conducted by the National Reference Center for Gastroenteritis Viruses in Dijon, France. Data on the predominant circulating strains are available going back to 1997.

### Winter season definition

In the present study, the winter season was defined as the earliest start week and the latest end week of the 25 past winter AG epidemics in France observed by the Sentinelles network, resulting in a 22-week period from week 47 to week 16 of the following year. Past epidemics were determined using the epidemic thresholds method used by the Sentinelles network, which is based on a periodic regression model [22,23].

### Statistical analyses

Cumulative incidence rates of GP visits for winter AG were calculated by summing up weekly incidence rates over each winter of the study period. For ED visits, the proportion of the weekly number of ED visits for AG over the total number of ED visits was calculated for the full series and for each winter period. This indicator was considered more robust to change in data collection conditions, e.g. number of enrolled EDs, number of all-cause visits and proportion of visits with a coded diagnosis. For drug reimbursement data, weekly incidence was provided for the full series and a cumulative winter incidence rate was calculated.

A Pearson correlation coefficient was calculated to assess correlations between data sources. The correlation between each source of data was studied: ED visits vs drug reimbursement, ED visits vs GP visits and GP visits vs drug reimbursement. The correlation between each source of data and predominant circulating strains of norovirus and rotavirus was studied using the non-parametric Kruskal–Wallis test. These correlations were calculated over the full series, including non-winter season data.

For winter seasons, incidence data and proportion data were described by the median of first and third quartiles (Q1; Q3), as this is a robust method for studying dispersion when data do not fit the normal assumptions. A generalised additive model using a Poisson law was applied to analyse the variation of tendency for the three datasets. The offset terms were population and age group defined as 0­–4 years, 5­–14, years, 15–64 years and 65 years and older. The outcome term for each winter period was annual incidence of GP visits, annual proportion of AG visits to ED visits and monthly incidence of drug reimbursement data. A spline component was added to model a nonlinear tendency. We computed this tendency considering different terms of tendency: common for all age groups and stratified by age group. The estimated value of the spline term was used to represent the changes in trends. The spline term allows evaluation of trends according to time and adjusted for population and age group. In order to test the hypothesis of geographical differences, AG cumulative incidence rate for GP visits were calculated for each winter season according to the five geographical regions defined by the telephone codes (01: Île-de-France; 02: North-West; 03: North-East; 04: South-East; 05: South-West). Similarly, cumulative incidence rate for winter periods of influenza-like illness and chickenpox for GP visits were computed to verify if the same tendency was observed for other diseases monitored by the Sentinelles network.

All analyses were performed using MASS and mgcv packages from GNU R software, version 3.1.1 [24].

## Results

### Winter incidence of acute gastroenteritis

During the 25 winter periods covered by the study, GP visit data showed a median weekly incidence rate of GP visits for AG of 173 cases per 100,000 inhabitants [Q1: 131; Q3: 256]. It ranged from 95 per 100,000 (1991/92 season) to 257 per 100,000 (2008/09 season). The cumulative incidence rate ranged from 2,403 per 100,000 inhabitants (1991/92 season) to 6,466 (2008/09 season) (Table).

**Table:** Incidence rates of general practitioner visits and proportion of hospital emergency department visits for winter acute gastroenteritis, Sentinelles network (1991/92 to 2014/15 winters), OSCOUR network (2004/05 to 2014/15 winters) and drug reimbursement data (2009/10 to 2014/15 winters), France

Winter season	Sentinelles network GP visits for AG, incidence per 100,000			OSCOUR network Proportion of ED visits for AG in %			Drug reimbursement AG incidence per 100,000		
	Cumulative incidence	Weekly incidence	Median (Q1–Q3)	Cumulative proportion	Weekly proportion	Median (Q1–Q3)	Cumulative incidence	Weekly incidence	Median (Q1–Q3)
1991/92	2,403	95	83–138	NA	NA	NA	NA	NA	NA
1992/93	3,289	124	83–158	NA	NA	NA	NA	NA	NA
1993/94	3,086	126	91–163	NA	NA	NA	NA	(NA	NA
1994/95	3,700	132	111–239	NA	NA	NA	NA	NA	NA
1995/96	3,541	139	104–221	NA	NA	NA	NA	NA	NA
1996/97	3,628	117	100–224	NA	NA	NA	NA	NA	NA
1997/98	3,673	139	102–216	NA	NA	NA	NA	NA	NA
1998/99	4,509	174	154–247	NA	NA	NA	NA	NA	NA
1999/00	4,692	157	134–258	NA	NA	NA	NA	NA	NA
2000/01	6,240	199	157–354	NA	NA	NA	NA	NA	NA
2001/02	3,920	145	124–231	NA	NA	NA	NA	NA	NA
2002/03	5,583	211	153–311	NA	NA	NA	NA	NA	NA
2003/04	5,090	210	177–278	NA	NA	NA	NA	NA	NA
2004/05	5,421	225	172–289	3.0	3.5	2.1–3.8	NA	NA	NA
2005/06	5,515	234	159–293	3.0	3.0	2.1–3.9	NA	NA	NA
2006/07	4,821	166	137–271	2.7	2.6	2.2–3.1	NA	NA	NA
2007/08	5,971	243	161–357	2.8	2.6	2.2–3.2	NA	NA	NA
2008/09	6,466	257	233–350	2.7	2.7	2.2–3.0	NA	NA	NA
2009/10	6,445	247	175–352	2.5	2.8	2.0–3.1	4,962	297	266–445
2010/11	4,489	177	148–227	2.3	2.4	1.8–2.6	6,310	252	204–356
2011/12	4,000	160	132–236	2.3	2.4	2.2–2.5	5,725	242	223–317
2012/13	4,586	184	157–227	2.3	2.3	2.1–2.5	6,015	264	206–334
2013/14	3,787	161	146–198	1.9	1.9	1.8–2.0	5,239	219	193–295
2014/15	3,918	166	147–216	2.2	2.3	2.1–2.5	4,841	214	186–254

AG: acute gastroenteritis; ED: emergency department; GP: general practitioner; NA: not available: OSCOUR: Organisation de la Surveillance COrdonnée des URgences.

ED visit data showed a median proportion of weekly ED visits for AG of 2.4% (Q1: 2.0%; Q3: 3.0%) during the 2005–2015 winters, ranging from 1.9% in 2013/14 to 3.5% in 2004/05. The winter proportions of the cumulative number of hospital ED visits ranged from 1.9% (2013/14 season) to 3.0% (2004/05 and 2005/06 seasons) (Table).

Drug reimbursement data from 2010 to 2015 showed a median weekly incidence rate of 246 per 100,000 inhabitants (Q1: 201; Q3: 309). It ranged from 214 per 100,000 (2014/15 season) to 297 per 100,000 (2009/10 season). The winter cumulative incidence rate ranged from 4,841 per 100,000 inhabitants (2014/15 season) to 6,310 (2010/11 season) (Table).

### Correlation between all data sources

**Figure 1:**
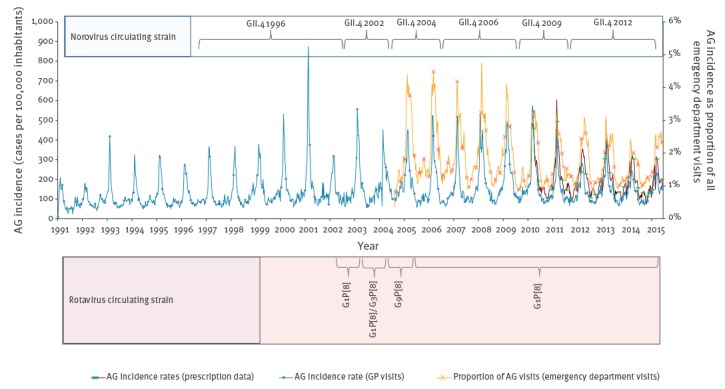
Weekly incidence rates of acute gastroenteritis general practitioner visits, drug reimbursement data, weekly proportion of emergency department visits for acute gastroenteritis, and predominant circulating strain of rotavirus and norovirus (National Reference Center for Gastroenteritis Viruses), France, 1991–2015

The three time series of AG surveillance data are represented in Figure 1. The correlation coefficients between data sources were: (i) 0.79 between GP visits and ED visits; (ii) 0.91 between GP visits and drug reimbursement data; and (iii) 0.76 between ED visits and drug reimbursement data (Figure 1). All correlations were statistically significant (p < 0.0001).

Since the emergence of norovirus GII.4 2012 in 2011/2012, incidences reported by GP visit and proportion of AG visits reported by ED were lower compared with the previous years when other norovirus GII.4 variants circulated (p < 0.0001). Similarly, the proportion of ED visits for AG was lower when rotavirus G1P [8] was the predominant circulating strain compared with rotavirus G9P [8] (p < 0.0001). This was not observed for GP visit data. No association has been found between drug reimbursement data and predominant circulating strains of norovirus, and the available data did not allow appraisal of the effect of the turnover in rotavirus strains (Figure 1).

### Trends in incidence of acute gastroenteritis during winter periods

**Figure 2:**
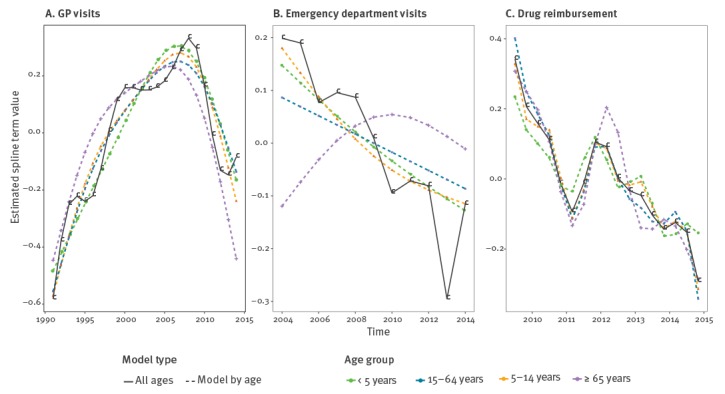
Acute gastroenteritis trends for general practitioner visits data, emergency department visits and drug reimbursement data using the estimated spline term value of a generalised additive model, France, 1991/1992 to 2014/2015 season

**Figure 3:**
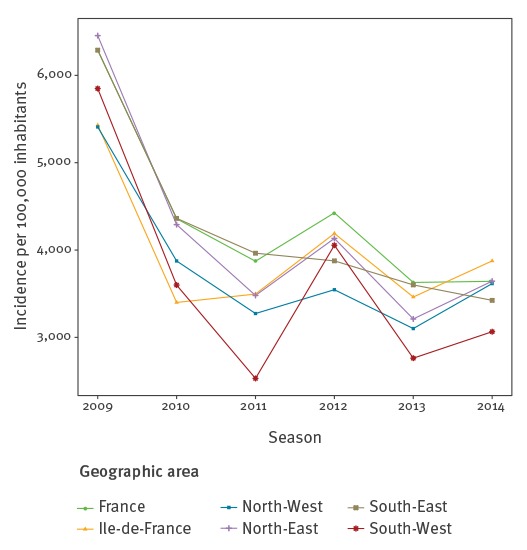
Cumulative incidence rates of general practitioner visits for winter acute gastroenteritis, by geographic area, Sentinelles network, France, 2009/10 to 2014/15

**Figure 4:**
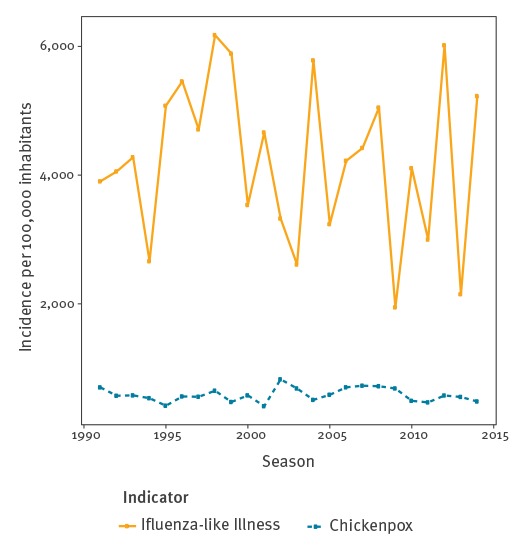
Cumulative incidence rates of general practitioner visits for winter influenza-like illness and chickenpox, Sentinelles network, France, 1991/1992 to 2014/2015 season

The trend in GP visits for AG increased in all age groups from the start of the study period in 1991 until the winter period 2008/09, and then decreased steadily in all age groups in the following years according to the estimated spline term value (Figure 2). The same tendency was observed in each of the five geographical areas (Figure 3). This decreasing trend was not noticed for other diseases monitored by the Sentinelles network, such as chickenpox or influenza-like illness (Figure 4). For ED visit data, a decreasing tendency was observed from 2004/05 until 2014/15, except for the age group 65 years old and over, for whom there was an increase until 2010 followed by a decrease (Figure 2). Drug reimbursement data also showed a decreasing trend in all age groups since the beginning of data collection in 2010 (Figure 2).

## Discussion

AG incidence in France almost doubled during the first 20 years of surveillance but has steadily decreased since 2009. Analyses performed on three independent datasets showed a similar decreasing trend for AG activity during the latest years of the study period, suggesting that this trend is not an artefact. There is no clear explanation for this decreasing trend and we will discuss the different hypothesis that could explain this phenomena.

Several studies have shown a reduction in AG incidence in children after a national rotavirus vaccine programme was implemented [11,12]. In 2014, in Europe, nine countries had already implemented such a programme: Austria, Belgium, Croatia, Finland, Germany, Greece, Luxembourg, Norway and the UK [10]. In France, although the rotavirus vaccination is not recommended routinely, the vaccine is available and it has been estimated that vaccine coverage for infants is between 5% and 9% [25]. A study in the Netherlands reported a decrease in rotavirus detection and GP consultations for AG in 2013/14, although no national rotavirus vaccine programme had been launched in this country [13]. The authors hypothesised that this decrease could be related to the rotavirus vaccination programmes in other European countries, to a less important rotavirus epidemic, or to a lower rate of transmission. It was later confirmed that this decrease was the result of a genuine drop in rotavirus circulation, as during the following season (2014/15), rotavirus epidemiology followed the usual pattern in the Netherlands (i.e. pre-2013/14 pattern). Thus the hypothesis of herd immunity resulting from the implementation of rotavirus vaccination policy in neighbouring countries was very unlikely [26]. In France, as in the Netherlands, it is unlikely that herd immunity resulting from immunisation and from those of the neighbouring countries contributed to the reduction in the incidence of AG, since we have shown a decrease in all age groups, including adults, for whom rotavirus accounts for only 1.4% of cases [5]. Also, in the adult population, the effect of rotavirus is likely to be negligible to the overall results of AG incidence [5]. Finally, we could not transpose our results to national surveillance data on norovirus because there is no systematic collection or continuous virological surveillance of AG cases in France.

It has also been hypothesised that a milder winter season and a decreasing birth rate could have contributed to decreased AG incidences [13]. Nevertheless, no gap between the observed winter temperature during the study period and the average historical winter temperature reported by Météo France during the last winter season was observed [27].

The predominant circulating strains of norovirus were correlated with GP visit data and ED visit data. Surprisingly, the emergence of the norovirus GII.4 2009 in 2009/10 and the GII.4 2012 variant in 2011/12 were temporally associated with a decreased incidence, whereas we could have expected an increase with emergence of a new variant in a naïve population. The predominant circulating variant of norovirus that is responsible for most of the cases in adults changes regularly. Since the 1990s, the emergence of several GII.4 variants has been reported worldwide [28,29]. Mutations and homologous recombination have been proposed as mechanisms driving the epochal evolution of GII.4, with the emergence of new variants in 1–3-year intervals causing global epidemics [30,31]. Since 2009, the GII.4 2009 and the GII.4 2012 variants have been the predominant circulating strains. It may be that the pathogenicity of these strains was less virulent than others or that the population has gained immunity to the GII.4 2012 variant since 2012 [32]. Indeed it is known that with the emergence of a new variant, for which there is no herd immunity, AG epidemic activity is higher [33]. However, there is no evidence that mortality and morbidity resulting from GII.4 2012 norovirus infection is different from those resulting from non-GII.4 2012 infections [34,35]. Thus, there are probably factors other than norovirus variant predominance that contribute to the incidence of AG during the winter season such as host susceptibility to other virus, levels of herd immunity, virulence of circulating viruses or public health interventions [36]. Indeed, norovirus and rotavirus bind to histo-blood group antigens which is controlled by FUT2 gene. A recent study showed that host with modified FUT2 gene were less likely to be infected with norovirus or rotavirus than those with non-modified FUT2 gene. An increase of the proportion of non-secretors in the French population could therefore result in a decrease of the number of AG cases. However no recent data are available in France to confirm this hypothesis [37].

Decreases in AG incidence may also be explained by behavioural modifications in the population. In 2009, the pandemic influenza epidemic may have had an impact on hygiene measures used by the population. Indeed, handwashing and the use of alcohol-based hand sanitisers during the epidemic period was strongly promoted and probably remained more frequent following the epidemic [38]. It has been shown that handwashing and the use of alcohol-based hand sanitisers were effective in reducing AG transmission [39,40]. Other behavioural modification, such as a shift towards private consulting or self-medication could explain this phenomenon. However, there was no change in the French health system that could have resulted in a significant shift of consulting patterns or drug deliveries. Indeed, in France, during the 2009–2015 period, the total number of GP consultations was stable [41]. Similarly, a study on over-the-counter drug sales for AG displayed no particular trends between 2008 and 2013 [42]. Thus, a shift in consulting habits or use of self-medication could not explain the observed trends.

This study has several limitations. First, for GP visit data, the incidence of AG could have been underestimated due to under-reporting from general practitioners omitting some cases. In contrast, it is also possible that the incidence has been overestimated because of misclassification by general practitioners who report cases that do not fit with the network definition. However, case definition, quality control of the collected data and analysis procedures remained stable since the beginning of AG surveillance. Thus, even if it is not possible to exclude under- or over-reporting, its impact on the trends would be very limited due to the stability of the Sentinelles surveillance network. Second, the representativeness of GPs from the Sentinelles network is questionable. Sentinelles GPs are more likely to be male and to have a higher number of consultations per week than other French GPs, but consultation estimates were weighted to overcome this bias, as already shown [15]. Third, drift in declaration practice of GPs may have occurred over time that could explain the observed tendency. This is unlikely because this phenomenon was not observed for other indicators monitored by the Sentinelles network, such as influenza-like illness or chickenpox (Figure 4). Additionally no major changes in methods used to estimate the incidence or in the participation of GPs occurred during the study period. Fourth, ED data may also suffer from inadvertent omissions, coding or diagnostic errors or physician noncompliance with the coding system. However, such potential biases, if they exist, would probably be stable over time and should not impact long-term tendencies, the more so that the definition used for AG and the analysis methods remained stable during the monitored period. Fifth, for the monitoring of AG, the Sentinelles network uses the number of acute diarrhoeal cases, despite the fact that vomiting can be an isolated symptom of gastroenteritis [43]. However, isolated vomiting is not the predominant clinical presentation of AG. Moreover, as the surveillance system was stable over time, it is unlikely that not considering episodes of vomiting without diarrhoea could have influenced the estimation of epidemic tendency, which was the main objective of this paper.

The reasons for the decreasing epidemiological trends of AG observed in this study remain unknown and are likely to be a multifactorial phenomenon. It justifies maintaining continuous surveillance of AG and strain typing of novel norovirus and rotavirus strains to describe secular trends in AG incidence. Such trends should be taken into account when interpreting the epidemiological changes following novel immunisation strategies against viral AG.

## References

[1] Majowicz SE Hall G Scallan E Adak GK Gauci C Jones TF A common, symptom-based case definition for gastroenteritis. Epidemiol Infect. 2008;136(7):886-94. 10.1017/S0950268807009375 17686196 PMC2870876

[2] Boschi-Pinto C Velebit L Shibuya K Estimating child mortality due to diarrhoea in developing countries. Bull World Health Organ. 2008;86(9):710-7. 10.2471/BLT.07.050054 18797647 PMC2649491

[3] Van Cauteren D De Valk H Vaux S Le Strat Y Vaillant V Burden of acute gastroenteritis and healthcare-seeking behaviour in France: a population-based study. Epidemiol Infect. 2012;140(4):697-705. 10.1017/S0950268811000999 21676346

[4] Huet F Chouchane M Cremillieux C Aubert M Caulin E Pothier P [Prospective epidemiological study of rotavirus gastroenteritis in Europe (REVEAL study). Results in the French area of the study]. Arch Pediatr. 2008;15(4):362-74. 10.1016/j.arcped.2008.01.021 18396016

[5] Arena C Amoros JP Vaillant V Ambert-Balay K Chikhi-Brachet R Jourdan-Da Silva N Acute diarrhea in adults consulting a general practitioner in France during winter: incidence, clinical characteristics, management and risk factors. BMC Infect Dis. 2014;14(1):574. 10.1186/s12879-014-0574-4 25358721 PMC4220050

[6] Chikhi-Brachet R Bon F Toubiana L Pothier P Nicolas JC Flahault A Virus diversity in a winter epidemic of acute diarrhea in France. J Clin Microbiol. 2002;40(11):4266-72. 10.1128/JCM.40.11.4266-4272.2002 12409408 PMC139722

[7] de Wit MA Koopmans MP Kortbeek LM van Leeuwen NJ Bartelds AI van Duynhoven YT Gastroenteritis in sentinel general practices,The Netherlands. Emerg Infect Dis. 2001;7(1):82-91. 10.3201/eid0701.010113 11266298 PMC2631671

[8] Karsten C Baumgarte S Friedrich AW von Eiff C Becker K Wosniok W Incidence and risk factors for community-acquired acute gastroenteritis in north-west Germany in 2004. Eur J Clin Microbiol Infect Dis. 2009;28(8):935-43. 10.1007/s10096-009-0729-1 19319582 PMC2723666

[9] World Health Organization (WHO). Global and national estimates of deaths under age five attributable to rotavirus infection: 2004. Geneva: WHO. 31 Mar 2006. Available from: http://www.who.int/immunization/monitoring_surveillance/burden/estimates/rotavirus/Global_national_estimates_2004_deaths_under_age_five_attributable_to_rotavirus_infection_2004.pdf?ua=1

[10] Parez N Giaquinto C Du Roure C Martinon-Torres F Spoulou V Van Damme P Rotavirus vaccination in Europe: drivers and barriers. Lancet Infect Dis. 2014;14(5):416-25. 10.1016/S1473-3099(14)70035-0 24758998

[11] Bawa Z Elliot AJ Morbey RA Ladhani S Cunliffe NA O’Brien SJ Assessing the Likely Impact of a Rotavirus Vaccination Program in England: The Contribution of Syndromic Surveillance. Clin Infect Dis. 2015;61(1):77-85. 10.1093/cid/civ264 25828997

[12] Aliabadi N Tate JE Haynes AK Parashar UD Centers for Disease Control and Prevention (CDC) Sustained decrease in laboratory detection of rotavirus after implementation of routine vaccination—United States, 2000-2014. MMWR Morb Mortal Wkly Rep. 2015;64(13):337-42. 25856253 PMC4584623

[13] Hahné S Hooiveld M Vennema H van Ginkel A de Melker H Wallinga J Exceptionally low rotavirus incidence in the Netherlands in 2013/14 in the absence of rotavirus vaccination. Euro Surveill. 2014;19(43):20945. 10.2807/1560-7917.ES2014.19.43.20945 25375899

[14] Haut Conseil de la Santé Publique (HCSP). Avis relatif à la vaccination des nourrissons vis-à-vis des gastroentérites à rotavirus. [Notice regarding vaccination of infants for rotavirus gastroenteritis]. Paris: HCSP; 21 Apr 2015. French. Available from: http://www.hcsp.fr/Explore.cgi/Telecharger?NomFichier=hcspa20150421_rotavirussusprecovaccnourrisson.pdf

[15] Souty C Turbelin C Blanchon T Hanslik T Le Strat Y Boëlle PY Improving disease incidence estimates in primary care surveillance systems. Popul Health Metr. 2014;12(1):19. 10.1186/s12963-014-0019-8 25435814 PMC4244096

[16] Blanchon T. Web-based sentinel provider surveillance network in France. In: M'Ikanatha NM, Lynfield R, Beneden CAV, Valk Hd, editors. Infectious Disease Surveillance: John Wiley & Sons Ltd; 2013. p. 418-25.

[17] Flahault A Blanchon T Dorléans Y Toubiana L Vibert JF Valleron AJ Virtual surveillance of communicable diseases: a 20-year experience in France. Stat Methods Med Res. 2006;15(5):413-21. 10.1177/0962280206071639 17089946

[18] Caserio-Schönemann C Meynard JB Ten years experience of syndromic surveillance for civil and military public health, France, 2004-2014. Euro Surveill. 2015;20(19):35-8. 10.2807/1560-7917.ES2015.20.19.21126 25990360

[19] World Health Organization (WHO). International Statistical Classification of Diseases and Related Health Problems 10th Revision 2016. Geneva: WHO. [Accessed 2 Feb 2016]. Available from: http://apps.who.int/classifications/icd10/browse/2016/en

[20] Tuppin P de Roquefeuil L Weill A Ricordeau P Merlière Y French national health insurance information system and the permanent beneficiaries sample. Rev Epidemiol Sante Publique. 2010;58(4):286-90. 10.1016/j.respe.2010.04.005 20598822

[21] Bounoure F Beaudeau P Mouly D Skiba M Lahiani-Skiba M Syndromic surveillance of acute gastroenteritis based on drug consumption. Epidemiol Infect. 2011;139(9):1388-95. 10.1017/S095026881000261X 21108871

[22] Costagliola D Flahault A Galinec D Garnerin P Menares J Valleron AJ A routine tool for detection and assessment of epidemics of influenza-like syndromes in France. Am J Public Health. 1991;81(1):97-9. 10.2105/AJPH.81.1.97 1983924 PMC1404927

[23] Réseau Sentinelles. Historique des épidémies. [History of epidemics]. Paris: Réseau Sentinelles. [Accessed 02/2016]. French. Available from: http://websenti.u707.jussieu.fr/sentiweb/?page=epidemies

[24] R Development Core Team. A language and environment for statistical computing. Vienna: the R Foundation for Statistical Computing; 2014. Available from: http://www.R-project.org

[25] Haute autorité de santé. (HAS). Commission de la transparence. Avis. RotaTeq. Saint-Denis La Plaine Cedex: HAS; 1 Apr 2015. French. Available from: http://www.has-sante.fr/portail/upload/docs/evamed/CT-13560_ROTATEQ_PIC_INS_Avis3_CT13560.pdf

[26] Pijnacker R Mughini-Gras L Vennema H Duizer E Pelt W Marked Decrease in Rotavirus Detections Among Preschool Children Unvaccinated for Rotavirus in the Netherlands, 2014. Pediatr Infect Dis J. 2016;35(7):809-11. 10.1097/INF.0000000000001162 27097349

[27] Météo France. Climat passé et futur - bilans climatiques. [Past and future climate – overall climate]. Saint-Mande Cedex: Météo France; [Accessed 1 Jan 2016]. French. Available from: http://www.meteofrance.fr/climat-passe-et-futur/bilans-climatiques

[28] Siebenga JJ Vennema H Zheng DP Vinjé J Lee BE Pang XL Norovirus illness is a global problem: emergence and spread of norovirus GII.4 variants, 2001-2007. J Infect Dis. 2009;200(5):802-12. 10.1086/605127 19627248

[29] van Beek J Ambert-Balay K Botteldoorn N Eden JS Fonager J Hewitt J Indications for worldwide increased norovirus activity associated with emergence of a new variant of genotype II.4, late 2012. Euro Surveill. 2013;18(1):8-9. 23305715

[30] Bull RA Eden JS Rawlinson WD White PA Rapid evolution of pandemic noroviruses of the GII.4 lineage. PLoS Pathog. 2010;6(3):e1000831. 10.1371/journal.ppat.1000831 20360972 PMC2847951

[31] Siebenga JJ Vennema H Renckens B de Bruin E van der Veer B Siezen RJ Epochal evolution of GGII.4 norovirus capsid proteins from 1995 to 2006. J Virol. 2007;81(18):9932-41. 10.1128/JVI.00674-07 17609280 PMC2045401

[32] Debbink K Lindesmith LC Donaldson EF Costantini V Beltramello M Corti D Emergence of new pandemic GII.4 Sydney norovirus strain correlates with escape from herd immunity. J Infect Dis. 2013;208(11):1877-87. 10.1093/infdis/jit370 23908476 PMC3814837

[33] Lee BE Pang XL New strains of norovirus and the mystery of viral gastroenteritis epidemics. CMAJ. 2013;185(16):1381-2. 10.1503/cmaj.130426 24003105 PMC3826359

[34] Leshem E Wikswo M Barclay L Brandt E Storm W Salehi E Effects and clinical significance of GII.4 Sydney norovirus, United States, 2012-2013. Emerg Infect Dis. 2013;19(8):1231-8. 10.3201/eid1908.130458 23886013 PMC3739516

[35] Allen DJ Adams NL Aladin F Harris JP Brown DW Emergence of the GII-4 Norovirus Sydney2012 strain in England, winter 2012-2013. PLoS One. 2014;9(2):e88978. 10.1371/journal.pone.0088978 24551201 PMC3923861

[36] Hasing ME Lee BE Preiksaitis JK Tellier R Honish L Senthilselvan A Emergence of a new norovirus GII.4 variant and changes in the historical biennial pattern of norovirus outbreak activity in Alberta, Canada, from 2008 to 2013. J Clin Microbiol. 2013;51(7):2204-11. 10.1128/JCM.00663-13 23637302 PMC3697663

[37] Kambhampati A Payne DC Costantini V Lopman BA Host Genetic Susceptibility to Enteric Viruses: A Systematic Review and Metaanalysis. Clin Infect Dis. 2016;62(1):11-8. 10.1093/cid/civ873 26508510 PMC4679673

[38] Van Cauteren D Vaux S de Valk H Le Strat Y Vaillant V Lévy-Bruhl D Burden of influenza, healthcare seeking behaviour and hygiene measures during the A(H1N1)2009 pandemic in France: a population based study. BMC Public Health. 2012;12(1):947. 10.1186/1471-2458-12-947 23127166 PMC3508974

[39] de Wit MA Koopmans MP van Duynhoven YT Risk factors for norovirus, Sapporo-like virus, and group A rotavirus gastroenteritis. Emerg Infect Dis. 2003;9(12):1563-70. 10.3201/eid0912.020076 14720397 PMC3034344

[40] Prazuck T Compte-Nguyen G Pelat C Sunder S Blanchon T Reducing gastroenteritis occurrences and their consequences in elementary schools with alcohol-based hand sanitizers. Pediatr Infect Dis J. 2010;29(11):994-8. 21046699

[41] Base Sniiram (Système National D’Information Inter-Régimes De L’Assurance Maladie). Caisse Nationale d’Assurance Maladie des Travailleurs Salariés. [French National Health Insurance Fund for Employees]. France: CNAMTS. [Accessed May 2016]. French. Available from: https://www.ameli.fr/l-assurance-maladie/statistiques-et-publications/sniiram/finalites-du-sniiram.php

[42] Pivette M Mueller JE Crépey P Bar-Hen A Surveillance of gastrointestinal disease in France using drug sales data. Epidemics. 2014;8:1-8. 10.1016/j.epidem.2014.05.001 25240898

[43] Kirby AE Streby A Moe CL Vomiting as a Symptom and Transmission Risk in Norovirus Illness: Evidence from Human Challenge Studies. PLoS One. 2016;11(4):e0143759. 10.1371/journal.pone.0143759 27116105 PMC4845978

